# The Addition of Co into CuO–ZnO Oxides Triggers High Antibacterial Activity and Low Cytotoxicity

**DOI:** 10.3390/nano13212823

**Published:** 2023-10-25

**Authors:** Elvira Maria Bauer, Alessandro Talone, Patrizia Imperatori, Rossella Briancesco, Lucia Bonadonna, Marilena Carbone

**Affiliations:** 1 Institute of Structure of Matter-Italian National Research Council (ISM-CNR), Via Salaria Km 29.3, 00015 Monterotondo, Italy; elvira.bauer@ism.cnr.it (E.M.B.); patrizia.imperatori@cnr.it (P.I.); 2 Department of Chemical Science and Technologies, University of Rome Tor Vergata, Via della Ricerca Scientifica, 00133 Rome, Italy; atalone84@gmail.com; 3 National Center for Water Safety, Italian National Health Institute, Viale Regina Elena 299, 00161 Rome, Italy; rossella.briancesco@iss.it (R.B.); lucia.bonadonna@iss.it (L.B.)

**Keywords:** CuO, ZnO, Co_3_O_4_, tenorite, solid solution, antibacterial efficacy, cytotoxicity

## Abstract

In the present work, a simple two-step method is proposed for mixed oxide synthesis aimed at the achievement of antibacterial nanomaterials. In particular, Cu, Zn and Co have been selected to achieve single-, double- and triple-cation oxides. The synthesized samples are characterized by XRD, IR, SEM and EDX, indicating the formation of either crystalline or amorphous hydrocarbonate precursors. The oxides present one or two crystalline phases, depending on their composition; the triple-cation oxides form a solid solution of tenorite. Also, the morphology of the samples varies with the composition, yielding nanoparticles, filaments and hydrangea-like microaggregates. The antibacterial assays are conducted against *E. coli* and indicate an enhanced efficacy, especially displayed by the oxide containing 3% Co and 9% Zn incorporated into the CuO lattice. The oxides with the highest antibacterial properties are tested for their cytotoxicity, indicating a low toxicity impact, in line with literature data.

## 1. Introduction

Antimicrobial resistance (AMR) is a global health and development risk that the World Health Organization (WHO) declared among the top 10 global public health threats facing humanity [1]. Misuse and overuse of antimicrobials, lack of clean water and sanitation and inadequate infection prevention and control are the main drivers in the development of drug-resistant pathogens. This increases the risk of infection during major surgeries and cancer treatments and consequent significant economic losses due to prolonged hospital stays and financial challenges impacting affected patients. New mechanisms of drug resistance have been developed by pathogens, leading to multi- and pan-resistant bacteria, also known as “superbugs”.

Control actions are being implemented to contain the problem, such as the Global Antimicrobial Resistance and Use Surveillance System (GLASS), as well as actionable surveillance implemented by the European Union to monitor sewage treatment plants serving more than 100,000 people for antibiotic resistance and environmental risks [2]. However, in terms of actions against AMR, the clinical pipeline of new antimicrobials is dying out. In 2019, WHO identified 32 antibiotics in clinical development that address the list of priority pathogens, only 6 of which were classified as innovative. Antimicrobials are meant to prevent and treat infections in humans, animals and plants; they can be selective against a single target or act against a broad spectrum of bacteria. Several strategies can be implemented to develop new antimicrobials, ranging from the employment of biocidal peptides of different origin (mammalian-, insect-, amphibian- and microorganism-derived) [3,4] to the development of new classes of antibiotics [5], the application of combinatory therapies [6] and the employment of nanomaterials [7,8,9]. The latter, in particular, display a versatility of applications since they can be embedded in clothes [10] and several types of matrices in varied contexts. For instance, they can be incorporated in water and air filters [11,12], dentistry fillings [13] and food packaging [14]. Furthermore, since they do not present the same mechanisms of action of standard antibiotics, they can be of extreme use against multi-drug-resistant (MDR) bacteria [15,16]. Types of nanomaterials and synthetic methods are crucial to determine their properties and, as a consequence, their efficacy in antimicrobial action. Metals and their supports [17,18,19,20,21,22,23,24,25,26,27,28,29] and metal oxide [30,31,32,33] nanoparticles (NPs), as well as carbon quantum dots [34,35,36,37,38,39,40], are known to effectively inhibit the growth of a wide range of sensitive and resistant Gram-positive and -negative bacteria, emerging as potential candidates to challenge antimicrobial resistance by modulated action [41]. Several metals and metal oxides have been extensively used as antimicrobials such as Fe_2_O_3_ [42], CuO and ZnO [43,44,45,46,47,48,49,50,51,52]. The use of Co_3_O_4_ instead represents a more recent trend, especially for bacteria remediation in water [53]. Furthermore, the lack of harm in vivo was verified for ZnO [54]. Antibacterial activity of cobalt compounds was investigated by Aguado et al. [55], who incorporated it into metal–organic frameworks. An enhancement of the metal oxide antimicrobial power has been explored through the employment of mixed oxides, where cations of different types are simultaneously present in the lattice and may have a synergistic effect on the antimicrobial activity. This is, for instance, the case in mixed oxides based on Cu–Zn–Mn [56], Zn–Mg–Cu [57] or metal/metal oxide systems [58,59]. The synthesis of the oxides can be carried out through several methods, including hydrothermal or solvothermal [60,61,62,63], sol–gel [64], phytosynthetic [65], via precipitation [66,67] and through innovative solvents [68]. However, in order to achieve mixed oxides, the application of the co-precipitation method is a preferential pathway to achieve homogeneous distribution of the cations into the “hosting” lattice (or a homogeneous solid solution). In this paper, we probe the biocide effects against *E. coli* of Co in a mixed oxide; therefore, we synthesized, characterized and comparatively investigated the antimicrobial properties of single-, double- and triple-cation mixed oxides, based on Cu, Zn and Co, as well as their cytotoxicity. The oxide samples were all synthesized using a simple two-step procedure and were subjected to structural and morphological characterization.

We found that oxides displaying a single crystalline phase, i.e., tenorite, are the most efficient as antimicrobials and, among them, the sample containing 3% cobalt is the most effective. Furthermore, the latter sample displays cytotoxicity in line with the other mixed oxides. The promising results we achieved against a Gram-positive bacterium, i.e., *E. coli*, open up new perspectives for testing Co-containing mixed oxides against a broader spectrum of bacteria, including Gram-negative ones, such as *S. aureus*.

## 2. Materials and Methods

### 2.1. Reagents and Equipment

Copper nitrate trihydrate (Cu(NO_3_)_2_·3H_2_O), zinc nitrate tetrahydrate (Zn(NO_3_)_2_·4H_2_O) and cobalt nitrate hexahydrate (Co(NO_3_)_2_·6H_2_O) were purchased from Fluka Chemicals (purity grade ≥ 99%). Sodium hydrogen carbonate (NaHCO_3_) was used as a base and was provided from Janssen Pharmaceutica Co., (Geel, Belgium) with a purity grade of ≥99.9%. The TSA plates and the granulated agar were prepared from Oxoid Culture Media & Reagents (Thermo Fisher Scientific Inc., Waltham, MA, USA) and Becton Dickinson Company (BD Life Sciences, Franklin Lakes, NJ, USA), respectively.

FT-IR spectra were recorded with a Shimadzu IRPrestige-21 apparatus, equipped with an ATR Specac Golden Gate single-reflection diamond in the 4000–400 cm^−1^ range. XRPD characterizations were performed on a Seifert 3003TT automatic diffractometer using Cu-Kα radiation (λ = 1.5418 Å) in the range 10 < 2θ < 80°. SEM images were taken using a FE-SEM, model SUPRA TM 35, Carl Zeiss SMT, Oberkochen, Germany, operated at 7 KV, equipped with Energy Dispersive Microanalysis (EDS/EDX, INCAx-sight, Model:7426, Oxford Instruments, Abingdon, Oxfordshire, UK), operating at 20 kV.

### 2.2. Synthetic Procedure

The synthesis of the oxides was carried out in two steps, i.e., the achievement of the precursors, followed by their calcination.

A total of nine precursors and nine oxides were synthesized, with different cation proportions, including single-cation reference compounds, double-cation and triple-cation compounds in different proportions, as listed in Table 1.

In a typical synthesis, 10 mmol of Cu(NO_3_)_2_·3H_2_O, Zn(NO_3_)_2_·4H_2_O and Co(NO_3_)_2_·6H_2_O in suitable proportions were dissolved in 150 mL of deionized water.

Upon addition of 60 mmol of NaHCO_3_ as powder, under magnetic stirring at room temperature, a light blue precipitate was immediately observed. The reaction vessel was sealed, and the suspension was kept under stirring for three hours to allow digestion in the presence of CO_2_. Typical reaction equations that may occur in the vessel are described in Equations (1) and (2).

2M(NO_3_)_2_ + 4NaHCO_3_ → M_2_CO_3_(OH)_2_ + 4NaNO_3_ + 3CO_2_ + H_2_O (1)

5Zn(NO_3_)_2_ + 10NaHCO_3_ → Zn_5_(OH)_6_(CO_3_)_2_ + 10NaNO_3_ + 8CO_2_ + 2H_2_O (2)

M may indicate a single cation, as well as a mixture of 2 or 3 cations. As for hydrogencarbonate, typical equations in a reaction vessel are described in Equations (3) through (5).

HCO_3_^−^ → CO_3_^2−^ + H^+^ (3)

HCO_3_^−^+ H_2_O → OH^−^ + H_2_CO_3_ (4)

H_2_CO_3_ → CO_2_ + H_2_O → CO_3_^2−^ + 2H^+^ (5)

The light blue-to-pale green precipitate was filtered, washed with deionized water and dried at 50 °C for 12 h. Finally, the precursors were calcined in a muffle furnace in air at 350 °C for 3 h, after heating, with a ramp of 2 °C per minute. Black powders were obtained for all samples, except for the ZnO single oxide, which was white instead.

### 2.3. Bacterial Growth Inhibition Assay

The bacterial growth inhibition assay was performed in a Petri dish test unit (90 mm × 16.2 mm) containing Tryptic Soy Agar (TSA, Thermo Scientific Oxoid, Thermo Fisher Scientific Inc., Waltham, MA, USA) culture medium at a specific concentration of mixed oxide nanoparticles (50, 100, 200, 400 µg/L). The nanoparticles were placed in glass tubes, to which 25 mL of melted Tryptic Soy Agar medium (agar 20 g/L) was added, sonicated (28–34 KHz) at 50 °C for 30 min and, finally, sterilized at 121 °C for 15 min. The solution was then poured into a test unit and immediately hardened in a freezer to avoid possible precipitation of nanoparticles.

The model organism used in this study was *Escherichia coli* (*E. coli* strain ATCC^TM^ 25922^TM^) acquired from Thermo Scientific Oxoid.

*E. coli* cultures were prepared overnight at 37 °C in the dark using Tryptic Soy Broth (TSB, Thermo Scientific Oxoid), and 100 μL of an appropriate dilution (equivalent to approximately 200 bacterial cells) was used to inoculate TSA agar Petri dishes with the specific concentrations of nanoparticles.

The dishes were then incubated at 37 °C in the dark. Each concentration of nanoparticles was prepared in three replicates. After 24 h of incubation, colony-forming units (CFU) were counted in each test unit. Agar media without nanoparticles were used as controls.

Growth inhibition was calculated using the formula

% growth inhibition = 100 − (N/N_0_ × 100)

where N and N_0_ are the colony-forming units (CFU) on the solid TSA agar medium with and without nanoparticles, respectively.

### 2.4. Cytotoxicity Assays

Human embryonic kidney 293 (HEK-293) cells cultured in Dulbecco’s Modified Eagle Medium (DMEM, Sigma Aldrich, St. Louis, MO, USA), supplemented with 2 mM glutamine and 10% Fetal Bovine Serum (FBS, Sigma), were maintained in humidified incubator at 10% CO_2_ and periodically tested to ensure the absence of mycoplasma contamination.

In the experiments performed to evaluate the toxicity of the metallic oxide nanoparticle, HEK-293 cells were seeded on 24-multiwell plates at a density of 105 cells/well and grown until confluence. Cells were then incubated for 24 h in the absence or in the presence of 50, 100, 200 and 400 μg/mL of metallic oxide nanoparticles suspended in DMEM and sonicated for 30 min (100 W, 30 kHz and 60% amplitude). Afterwards, cells were washed with PBS, and surviving cells were counted using the trypan blue method.

## 3. Results

Precursors and oxides underwent structural and morphological characterization followed by the investigation of the antimicrobial power of the oxides, as well as their cytotoxicity. In the following sections, the results are reported in subsections referring to precursor and oxide characterizations and microbiological assays.

### 3.1. Precursors

#### 3.1.1. XRD Patterns

**Figure 1:**
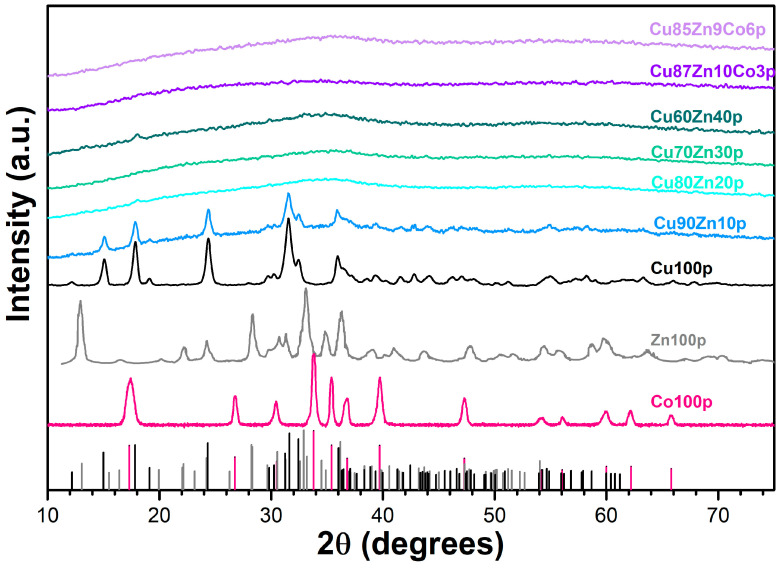
XRD pattern of the precursor samples. The vertical line corresponds to the reference malachite (solid black line), hydrozincite (solid gray line) and Co hydroxycarbonate (pink solid line).

The XRD measurements carried out on the precursors and reported in Figure 1 showed two types of patterns, i.e., crystalline phases, for single-cation precursors and for the double-cation Cu90Zn10, whereas all other samples were widely amorphous. In addition, three different crystalline phases are evidenced for the single-cation precursors. Their identification was carried out by inquiry of the JCPDS database and comparison to reference compounds. The phases malachite [69], hydrozincite [70] and cobalt hydroxycarbonate [71,72] were found for the samples Cu100p, Zn100p and Co100p, respectively, i.e., in all cases, hydroxycarbonate compounds.

The lack of crystallinity in the three-cation (Cu–Zn–Co) hydroxycarbonates was already observed to some extent [73] and can easily be related to the mismatch of ionic radii of the cations in forming solid solutions, as well as the preparation conditions. Samples in the present work were synthesized at room temperature by adding NaHCO_3_ as powder, whereas larger crystallinity may be achieved by preparing separate solutions of NaHCO_3_ and cation nitrates and preheating them at 65 °C before mixing them.

Cu and Zn hydroxycarbonate lattices are monoclinic, whereas Co hydroxycarbonate is orthorhombic, and the simultaneous presence of seeds of phases in different crystal systems may cause the overall amorphization. Precautions can be put in place to prevent the formation of amorphous compounds to a certain extent, though at the expense of simple operations.

As for the two-cation hydroxycarbonates, only the Cu90Zn10p is crystalline and retains the same malachite structure as in Cu100p, varying from previously reported synthetic procedures that exhibit crystallinity up to a Cu/Zn ratio of 70/30, always yielding a malachite structure [74]. In the present case, differences in the experimental conditions are related to (i) the concentration of the reagents, (ii) the way NaHCO_3_ is added to the solution (i.e., one-shot of powder) and (iii) the temperature of the reaction (room temperature vs. 65 °C).

#### 3.1.2. Infrared Spectra

**Figure 2:**
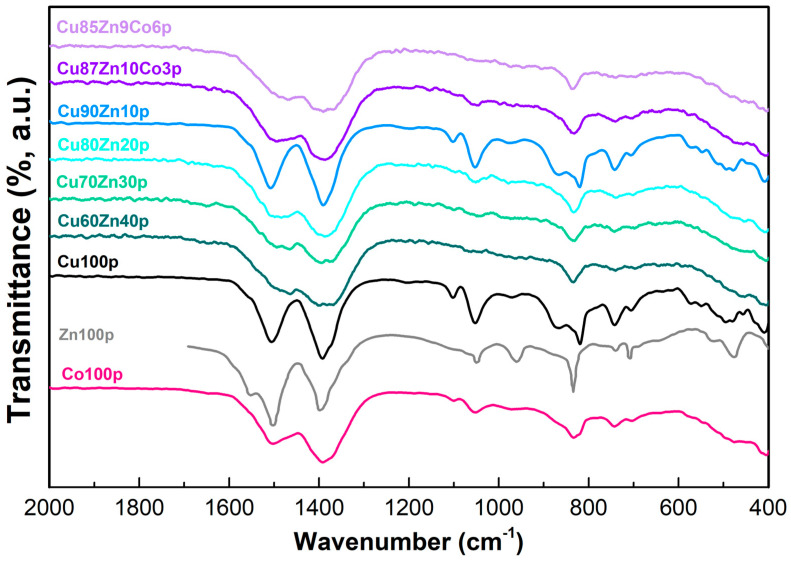
IR spectra of the synthesized precursors.

Due to the amorphous nature of some of the precursors, infrared spectroscopy was carried out to determine the type of compounds. The spectra are reported in Figure 2.

Numerous scientific papers are centered on the influence of precipitation conditions of Cu/Zn/Co nitrates with NaHCO_3_, which generally results in formation of different precursor hydroxycarbonates, focusing on the importance of temperature, concentration, duration of digestion and precipitation at constant, decreasing or increasing pH [75,76]. Most precipitation products are related to natural minerals such as malachite (Cu_2_(CO_3_)(OH)_2_), rosasite ((Cu,Zn)(CO_3_)(OH)_2_, where copper content is 33–50% and Zn content is 38%), zincian malachite (rosasite with low Zn content) or even the rare georgeite (Cu_5_(CO_3_)_3_(OH)_4_·6H_2_O) [77,78,79,80]. Quite often, the formation of synthetic rosasite with Zn contents higher than 10% leads to formation of aurichalcite (Zn_5_(OH)_6_(CO_3_)_2_) as a by-product [81]. Differences in their typical IR vibrations makes it possible to distinguish between the various classes of hydroxycarbonate precursors, even in the case of amorphous samples, where distinction by XRD powder diffraction is not possible.

All vibrations observed in our Co100p show the typical spectral features of a hydroxycarbonate-based material observed by XRD, i.e., Co(CO_3_)_0.5_(OH)·0.11 H_2_O, although most bands are considerably shifted in comparison to the data reported by Zhou et al. [82]. Indeed, no splitting of the asymmetric (1504 cm^−1^) and symmetric carbonate stretchings (1390 cm^−1^) were observed in the Co100p sample, but both bands were quite broad and shifted by nearly 50 cm^−1^ towards lower (asymmetric stretching) or higher wavenumbers (symmetric stretching). In particular, the feature assigned to the symmetric carbonate stretching, along with its shoulder around 1369 cm^−1^, is more in line with the signal typical for cobalt carbonate (1409 cm^−1^) [83]. Further broad carbonate vibrations are located at 833 cm^−1^ (*ν*_2_), 742 and 704 cm^−1^ (*ν*_4_), which characterize both hydroxycarbonates and carbonates. However, the presence of three weak signals at 1101, 1053 and 966 cm^−1^ sustains hydroxycarbonate as the only or, at least, a major component in Co100p. The spectral features of the sample Zn100p are identical to literature data for hydrozincite (Zn_5_(OH)_6_(CO_3_)_2_) [84], characterized by strong peaks at 1501 and 1400 cm^−1^ and a shoulder at 1551 cm^−1^ related to *ν*_3_ asymmetric C-O stretching. Interestingly the lower wavenumber asymmetric C-O stretching, generally a double peak around 1420 and 1383 cm^−1^ in hydrozincite, is not split in our case; rather, only one broad peak centered at 1400 cm^−1^ is present. Additional frequency splitting in this spectral region has been correlated with well-crystallized samples by Musić et al. [84]; therefore, the broadening and shifting towards higher wavenumbers is a clear sign of a low degree of crystallinity in our sample. Further peaks due to the presence of carbonate groups can be found at 1052 cm^−1^ (ν_1_), 834 cm^−1^ (*ν*_2_) and 708 cm^−1^ (*ν*_4_), while the -OH deformation band is located at 961 cm^−1^. Zn-O bond lattice stretching vibrations are observed at 517 and 476 cm^−1^. The spectrum of Cu100p closely resembles the vibrations characteristic for the mineral malachite. The assignment is confirmed especially by two strong bands at 1504 and 1393 cm^−1^ accompanied by weak shoulders that correspond to the splitting of the C-O asymmetric stretching *ν*_3_ in the region 1700 and 1300 cm^−1^ [77]. The broadening of the asymmetric stretching vibrations is most probably due to the poor crystallinity of the sample. OH deformation bands are observed at 1101, 1053 and 866 cm^−1^ in the Cu100p sample along with sharp *ν_2_* and *ν*_4_ O-C-O bendings around 820 and 743 cm^−1^ [77]. Characteristic but weak Cu-O stretching vibrations at 573 cm^−1^ further confirm the presence of a malachite-related molecular structure in the precursor sample. Substitution of Cu with high amounts of zinc (20–40%, i.e., samples Cu60Zn40p, Cu70Zn30p and Cu80Zn20p) in the malachite-based precursor material Cu100p leads to formation of a different precipitate whose FT-IR spectra resemble the features of zincian georgeite [80]. In all three samples, the asymmetric carbonate stretchings are broadened and slightly shifted towards lower wavenumbers, i.e., around 1495 and 1385 cm^−1^, and, especially in sample Cu60Zn40p, all hydroxyl related peaks are nearly absent. Both asymmetric signals likely derive from superimposition of carbonates bonded to copper and zinc. This is most evident in sample Cu70Zn30p, where four maxima at 1495, 1466, 1394 and 1369 cm^−1^ can be found. The intensity of hydroxyl- and carbonate-related bending modes around 1050 cm^−1^ and 750 cm^−1^ decrease with increasing zinc content, while the carbonate out-of-plane bending downshifts from 815 cm^−1^ towards 833 cm^−1^. Varying from this, the FT-IR spectrum of Cu90Zn10p (10% zinc substitution) is nearly indistinguishable from the pure malachite-related copper hydroxycarbonate sample Cu100p. All vibrational peaks are found at the same position as in the copper-based precursor material, thus indicating that the malachite structure has been maintained upon substitution with zinc. No sharp peaks around 1385 and 1050 cm^−1^ have been observed, ruling out contamination with hydroxynitrates [81,85,86].

Introduction of small amounts of cobalt (Cu87Zn10Co3p and Cu85Zn9Co6p) follow the same trend just observed for heavier substitution with zinc, i.e., isolation of georgeite-like precipitates. Indeed, both cobalt-substituted materials show only three broad vibration peaks, centered at 1493, 1386 and 833 cm^−1^, characteristic for zincian georgeite [80].

The isolation of zincian georgeite as the main precursor product is, to some extent, different from previous reports where georgeite-related compounds were only observed as a transient amorphous phase contaminated with hydroxynitrates [77]. Further transformation of georgeite into zincian malachite, auricalcite or Zn-rich amorphous materials upon room temperature synthesis followed by heating and aging in the mother liquor was also reported [86]. Stable zincian georgeite with a 2/1 and 1/1 copper/zinc ratio has been isolated by Kondrat et al. [78] when starting from copper acetate in a supercritical antisolvent precipitation process. Corresponding FT-IR spectra showed only three prominent bands in the region between 1600–700 cm^−1^ that were similar to our samples at the highest Zn content, thus proving that up to 50% Cu can be substituted by Zn in amorphous georgeite. Furthermore, Pollard et al. evidenced the high stability (up to two weeks at 20 °C) of Zn-rich georgeite in contact with the mother liquor, attributing the increased resistance to transformation in malachite-related structures to an increase of covalent metal–oxygen bonding associated with a decrease in unit cell parameters [80]. FT-IR analyses of our zinc-rich hydroxycarbonate precursor samples indicate that the synthesis parameters such as metal nitrate concentration, excess sodium bicarbonate and temperature adopted in our work seem to favor the formation of stable amorphous zincian georgeite.

### 3.2. Mixed Oxides

#### 3.2.1. XRD Patterns

**Figure 3:**
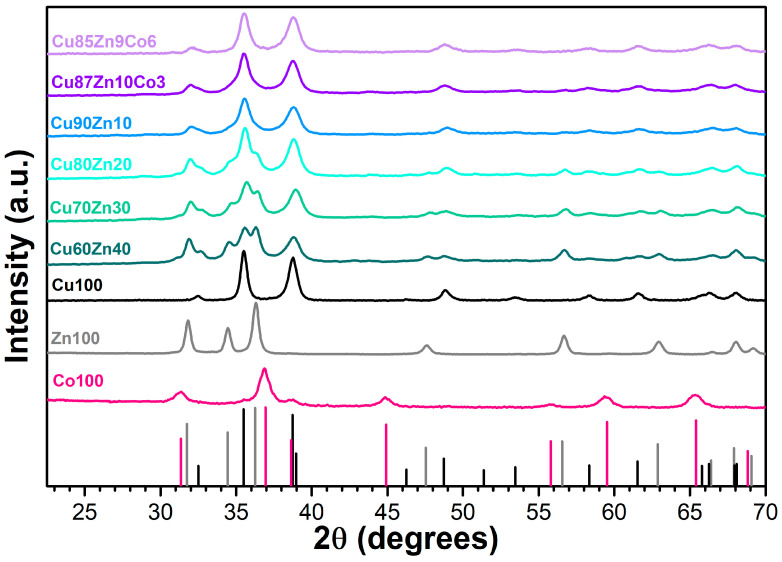
XRD pattern of single-, double- and triple-cation oxide samples. The vertical solid lines correspond to reference tenorite (solid black line), zincite (solid gray line) and spinel phases (pink solid line).

The XRD patterns of the oxides are reported in Figure 3, along with the reflexes of reference single oxide compounds, i.e., CuO with tenorite crystalline structure, monoclinic prismatic space group 2/m [87,88]; ZnO with zincite structure, hexagonal [89], space group P6_3_mc and Co_3_O_4_ with spinel structure, space group Fd-3m [90]. The samples Cu100, Zn100 and Co100 display a one-to-one correspondence with the reflexes of the reference compounds tenorite, zincite and spinel, respectively. The double- and triple-cation oxides were analyzed by Rietveld refinement to determine both the number of phases present, as well as the lattice parameters upon formation of solid solutions. The Cu90Zn10 is characterized by a single phase, i.e., tenorite, though with a variation in the lattice parameters and an increase in cell volume as compared to Cu100, compatible with 10% substitution of Zn in the CuO lattice [91]. Further increase in Zn content results in the segregation of a zincite phase, in percentages corresponding to 10.2%, 19.7% and 30.6% for the samples Cu80Zn20, Cu70Zn30 and Cu60Zn40, respectively. As for the lattice parameters, there is no significant variation in zincite in the binary cation oxides as compared to Zn100. Cu80Zn20, Cu70Zn30 and Cu60Zn40 have similar lattice parameters as Cu90Zn10, thus indicating that there is an upper solubility limit of Zn in tenorite corresponding to 10%, according to the current synthetic method. Beyond this level, zincite forms. However, although Cu^2+^ ions are present, they are hardly included into the zincite lattice, as indicated by the lattice parameters, which are nearly the same as in the sample Zn100.

It must be noted that the solubility of Zn in tenorite is preparation-dependent and that concentrations up to 30% of Zn can be accommodated into the tenorite lattice. These synthetic methods, however, require harsher conditions, lower concentrations and lower quantities, whereas, in the current study, we were opting for simple, smooth, low-temperature operations.

As for the triple cation oxide samples, the only phase present is tenorite, similar to the Cu90Zn10 sample. The atomic percentage of “guest” cations, i.e., the sum of Zn and Co in Cu87Zn10Co3 and Cu85Zn9Co6 is 13% and 15%, respectively. However, Co^2+^ has a slightly smaller ionic radius than Cu^2+^, differently from Zn^2+^, which is slightly larger. This allows the inclusion of a larger amount of guest cations into the tenorite lattice.

Lattice parameters, volumes and percentages of the various phases of the synthesized samples are reported in Table 2.

#### 3.2.2. SEM Images and EDX

**Figure 4:**
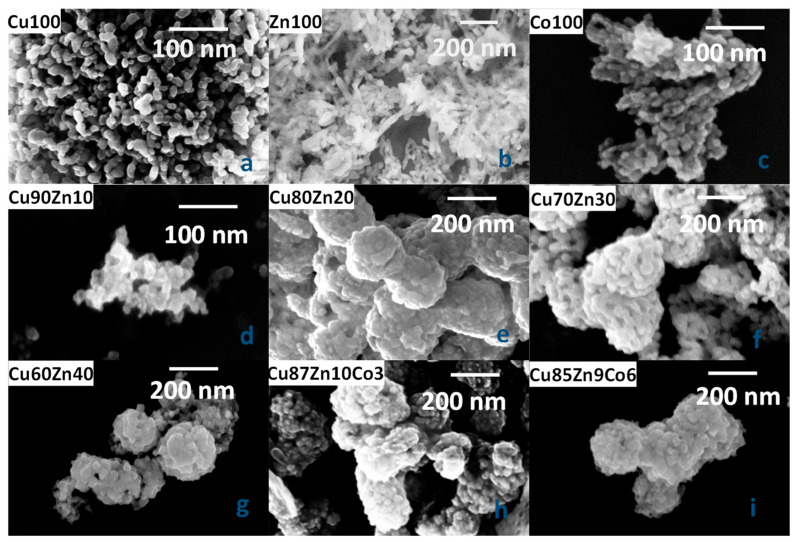
SEM images of the synthesized oxides: (**a**) sample Cu100, (**b**) sample Zn100, (**c**) sample Co100, (**d**) sample Cu90Zn10, (**e**) sample Cu80Zn20, (**f**) sample Cu70Zn30, (**g**) sample Cu60Zn40, (**h**) sample Cu87Zn10Co3, (**i**) sample Cu85Zn9Co6.

SEM images of the synthesized samples are shown in Figure 4. Single-cation oxides are sketched in the top raw (panels a, b and c), double-cation oxides are reported in panels d, e, f, and g and triple-cation oxides are in panels h and i.

Single-cation oxides display significantly different morphology, despite being synthesized in similar conditions. The synthetic procedure is a primary factor in determining the shape of the crystals. Recent tests conducted on CuO synthesis through different pathways, including sol–gel and precipitation methods as well as hydrothermal synthesis at different temperatures, with or without surfactant, pointed at the formation of different morphologies, including nanoparticles in the 20 nm range, forming microaggregates [88]. In the present investigation, we opted for synthetic conditions that would yield CuO nanoparticles in the tenths of nanometers range, thus selecting the precipitation method, followed by calcination at 350 °C, achieving corresponding morphologies and sizes. Once the preparation conditions are set for the first sample (CuO), all the other ones were synthesized by the same method. However, this does not yield the same shape for all samples. Previous studies on ZnO synthesis at low temperatures (40 °C), and a slight defect of base (NaOH), disclosed the formation of nanoparticles [92]. However, in the current setup, ZnO filaments formed, instead, with average diameter 50 ± 5 nm and average length 250 ± 15 nm. Spinel Co_3_O_4_ and the double-cation oxide Cu90Zn10 also form nanoparticles, with average diameter 20 ± 5 nm, whereas the remaining double-cation oxides all have the same morphology, with small differences in average dimensions. The dominant shapes are hydrangea-like agglomerates of diameters between 150–500 nm, formed by nanoparticles of 50 ± 10 nm. The average dimensions of the particles in the various samples are reported in Table 3.

The composition of the synthesized oxides was evaluated by EDX, and the results are reported in Table 4. The atomic percentages of metals and oxygen are very close to the nominal ones. The sum of residual N, Na and C is on average <0.2%, hence, close to the detection limit.

### 3.3. E. coli Viability

**Figure 5:**
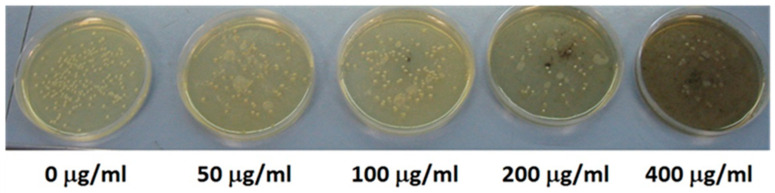
Petri dishes seeded with *E. coli* and added with different doses of Cu100. Blank control is on the left.

*E. coli* inhibition growth was assessed by CFU counting after 24 h incubation in the dark at four different oxide concentrations and compared to the control. In Figure 5, Petri dishes are reported after seeding with Cu100 (as an example) at the four selected concentrations, i.e., 50 μg/mL, 100 μg/mL, 200 μg/mL and 400 μg/mL, and 24 h incubation in the dark. The control is also reported.

**Figure 6:**
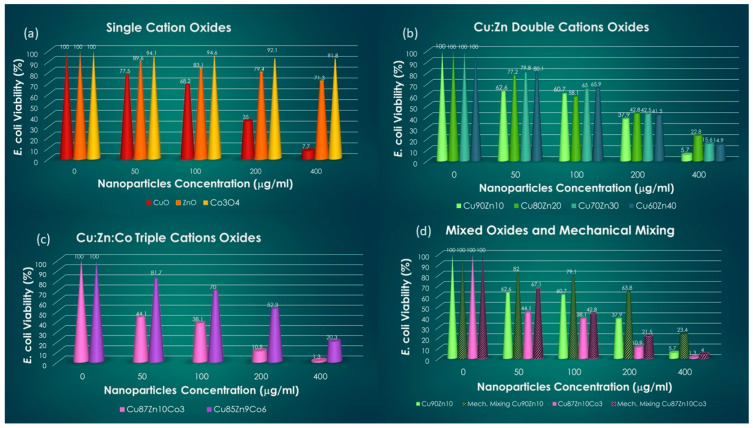
*E. coli* viabilty at various doses of oxides: (**a**) single-cation oxides, (**b**) double-cation oxides, (**c**) triple-cation oxides, (**d**) mechanical mixing of single oxides.

In Figure 6, the hystograms with the *E. coli* viability are reported in four different panels: (a) for single-cation oxides, (b) for double-cation oxides, (c) for triple-cation oxides and (d) for selected mixings of single oxides.

A general feature is shared by all the oxides, i.e., the growth inhibition is dose-dependent; hence, it is highest for the 400 μg/mL. The comparison among single oxides indicates a markedly stronger inhibition by CuO, especially at the highest dose, with a viability of 7.7%. This is in large contrast to the effect of Co_3_O_4_, which hardly inhibits *E. coli* growth, with a 91.8% viability at 400 μg/mL. It must be noted that the texture and size of CuO and Co_3_O_4_ nanoparticles is rather similar; therefore, the variation in effect is to be attributed to the type of metal and possibly to the crystal structure. ZnO has a kind of intermediate behavior, though, the morphology being different, this may play a role in the inhibition, in addition to the type of metal. Double-cation oxides retain the dose-dependency effect, thus displaying the highest inhibition at 400 μg/mL. Zn substitution in CuO has a positive effect in the sample Cu90Zn10, causing slightly lower *E. coli* viability than CuO. This can be attributed to a synergistic effect of Cu^2+^ and Zn^2+^ ions released in the medium in close contact. The other double-cation oxides have a mixed behavior, i.e., they are more effective than CuO at low doses (50 μg/mL and 100 μg/mL) and less effective at higher doses (200 μg/mL and 400 μg/mL). This type of behavior likely reflects the influence of the two distinct crystalline phases, the Zn-tenorite and the zincite, which may display different biocidal trends as a function of the concentration. Furthermore, the different morphology, as compared to Cu100 and Cu90Zn10, may also play a role. The presence of 3% Co in the triple-cation oxide has a dramatic effect on the *E. coli* viability, which is largely reduced with respect to CuO at all doses. In particular, it is already reduced to 44.1% at 50 μg/mL and is barely detectable at 400 μg/mL. A further increase in the Co percentage up to 6% is counterproductive and lowers the performance of Cu85Zn9Co6 below the level of Cu60Zn40. Since the mixed oxides display, on average, better performances than the single oxides, a test was carried out to determine whether the synergistic effect plays a role, i.e., whether the antimicrobial power is connected to the employment of mixed oxide nanoparticles, or if it is simply the simultaneous presence of cations in suitable proportion to determine the biocidal power. Therefore, single oxides were mixed in suitable proportions (mechanical mixtures), to achieve the same cation ratios as in the mixed oxides, and the viability tests were repeated in the analogous conditions. The outcome is reported in Figure 6d for the best-performing samples, i.e., Cu90Zn10 and Cu87Zn10Co3, at the highest doses. In both cases, the mechanical mixtures are less effective than the mixed oxides counterparts, thus indicating a positive correlation between mixed oxides and antimicrobial power.

### 3.4. Cytotoxicity Assays

**Figure 7:**
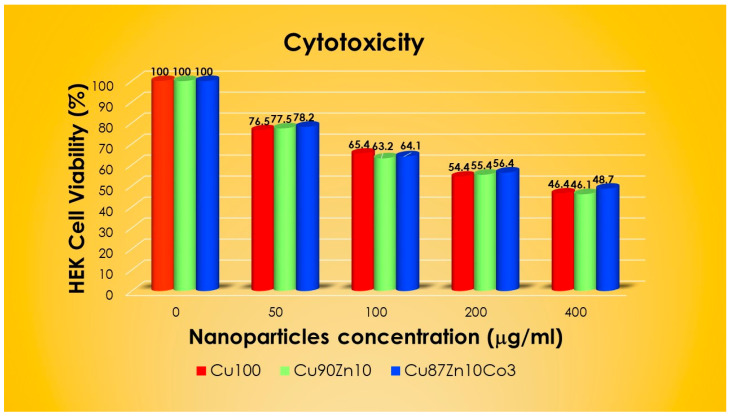
Cytotoxicity assay as a function of the doses of Cu100, Cu90Zn10 and Cu87Zn10Co3.

Cytotoxicity of the best-performing samples, i.e., Cu100, Cu90Zn10 and Cu87Zn10Co3, was determined by culturing 50, 100, 200 and 400 μg/mL of NPs with HEK 293 cells and counting the viable cells via a dye-exclusion test (trypan blue) after 24 h. The results, reported in Figure 7, indicate that all three oxides exhibit low cytotoxicity after exposure to the lowest dose (50 μg/mL) of the NP dispersion in DMEM, since the survivor rate is as high as 76–78%. For higher concentrations of NPs, cell viability decreased in a dose-dependent manner, reaching values below 50% at 400 μg/mL. There is no significant cytotoxicity difference among the three investigated samples and no clear relationship between the particle size and survival rate. Indeed, Cu100, characterized by the presence of spherical NPs of ca. 20 nm, behaves similarly to the bigger hydrogenea-like aggregates of Cu90Zn10 and Cu87Zn10Co3. This might be related to the actual aggregation of NPs in DMEM, which would cause a size homogenization, as already pointed out by several authors [93,94]. A rough estimation of the half inhibitory concentration (IC_50_) was made using the AAT Bioquest IC50 Calculator [95] and indicated doses in the range 50–100 μg/mL, i.e., 87 μg/mL for CuO100, 76 μg/mL for Cu90Zn10 and 68 μg/mL for Cu87Zn10Co3. The observed cytotoxicity is in line with literature data for CuO and ZnO NPs, summarized in Table 5. In more detail, cell viability tests after 24–48 h exposure to spherical CuO NPs issued IC_50_ values between 2.5 μg/mL and 65.5 μg/mL for HEK 293, Caco2, A459 and Balb/c 3T3 cell lines [93,96,97], whereas biofilm formation seems to lower cytotoxicity of the CuO NPs [98]. The IC_50_ of ZnO NPs with sizes 20–100 nm is on average slightly higher [99,100,101,102,103]. Ivask et al. [93] found overall higher cytotoxicity levels of CuO in a comparative study of CuO, ZnO and Co_3_O_4_ NPs of comparable sizes on Caco2, A 459 and Balb/c 3T3 cell lines. Cobalt oxide NPs, especially Co_3_O_4_ NPs, are often defined as inactive—although no cytotoxicity data are reported on HEK 293 cells (see Table 5)—due to their low degree of dispersion in the medium and consequent low concentration of free cobalt ions, i.e., one of the key factors for cobalt toxicity [93]. Cytotoxicity tests of Co, CoO and Co_3_O_4_ NPs on different cell lines showed that Co has higher toxicity in HBEC cells, and it is the only one that affects the growth of A549 cell lines [94]. Furthermore, Co_3_O_4_ NPs with dimensions above 20 nm were found to be considerably toxic only towards bronchial BEAS-2S cell lines [93,104]. On the contrary, Co_3_O_4_ NPs of a size of ~9 nm seem to show pronounced intracellularization (Trojan horse uptake) and ROS induction, thus leading to remarkable cytotoxicity for cells lines such as A459 [93]. The Cu87Zn10Co3 sample examined in our experiment does not exhibit higher cytotoxicity as compared to cobalt-free samples. Furthermore, the cytotoxic behavior of our double-cation sample Cu90Zn10 closely recalls pure CuO of the Cu100 sample. The cytotoxicity assessment of a similar Zn-substituted CuO (Cu_0.88_Zn_0.12_O), reported by Yuan et al. [105] on several cancer cells, indicated a dose-dependent apoptosis, while only feeble toxicity on HUVEC cells was observed. Inhibition of cell growth by the binary copper/zinc oxide has been related to ROS-mediated NF-κB activation [105], while, in the case of pure CuO NPs, other mechanisms seem to play a more important rule in ROS-mediated apoptosis [106]. However, a detailed analysis of the data on HUVEC and LO2 cells locates the inhibition rate around 20%, i.e., in line with the values detected in our samples Cu90Zn10 and Cu87Zn10Co3 (cell viability below 80% at the lowest exposure dose). Xu et al. [107] reported analogous results in using the same type of sample for Frog Embryo Teratogenesis tests. Further comparison with literature data for CuO and ZnO NPs on HEK 293 cells suggests a similar or even lower cytotoxicity in our experiments. For instance, Reddy and co-workers found a cell viability of approximately 60% upon exposure to 50 µg/mL spherical CuO NPs of 55 nm diameter [96]. In another investigation, incubation of HEK 293 cells with the same amount of spherical ZnO NPs (20–40 nm) resulted in cell viability values slightly above 80% [103]. Surprisingly, the three CuO-based samples investigated in our work were less cytotoxic than CuO particles examined in other works, thus indicating less propensity to ROS-mediated cell apoptosis towards HEK 293 cells.

## 4. Conclusions

In the present work, we synthesized Cu–Zn–Co-based oxides using a two-step procedure, the first phase being carried out at room temperature with the addition of powder NaHCO_3_, followed by calcination. Depending on the cations’ proportions, single-, double- and triple-cation oxides were obtained, which were fully characterized by spectroscopic and microscopic techniques. The synthetic methods cause the formation of hydroxycarbonates that are only crystalline in the case of the single-cation samples or low-Zn content double-cation ones. The subsequent calcination yields crystalline oxides. The single-cation oxides are tenorite, zincite and spinel phases. The mixed oxides, instead, present a single tenorite phase, in the case of double-cation oxides with low Zn content and triple-cation oxides. In all other cases, an additional zincite phase is observed. The Rietveld analysis of the XRD patterns suggests the inclusion of Zn and Co cations into the tenorite phase, whereas zincite does not include guest cations. The morphology of the oxides is also composition-dependent, since Cu100, Co100 and Cu90Zn10 samples are nanoparticles in the 20 nm diameter range, Zn100 is characterized by the formation of filaments and all other samples create hydrangea-like microaggregates. The antibacterial assays performed against *E. coli* clearly indicate a higher biocide power of the double- and triple-cation oxides. Particularly, the addition of Co into the tenorite lattice of Zn-tenorite allows the enhancement of the antibacterial properties against *E. coli*. Since the employment of Co is rather unusual, tests of cytotoxicity were performed and a thorough comparison with the literature was carried out to ensure the safety of our samples.

## Figures and Tables

**Table 1:** Naming of the synthesized compounds and % of each cation.

Precursor	Oxide	Cu	Zn	Co
Cu100p	Cu100	100	--	--
Zn100p	Zn100	--	100	--
Co100p	Co100	--	--	100
Cu90Zn10p	Cu90Zn10	90	10	--
Cu80Zn20p	Cu80Zn20	80	20	--
Cu70Zn30p	Cu70Zn30	70	30	--
Cu60Zn40p	Cu60Zn40	60	40	--
Cu87Zn10Co3p	Cu87Zn10Co3	87	10	3
Cu85Zn9Co6p	Cu85Zn9Co6	85	9	6

**Table 2:** Percentage of tenorite and zincite in the synthesized samples and corresponding lattice parameters. -- implies that the phase is not present. Same as other tables.

	Tenorite					Zincite			
	%	a (Å)	b (Å)	c (Å)	V (Å^3^)	%	a(Å)	c (Å)	V (Å^3^)
Sample									
Cu100	100	5.133	3.430	4.678	81.2	--			
Cu90Zn10	100	5.155	3.395	4.738	81.8	--			
Cu80Zn20	89.8	5.150	3.403	4.731	81.7	10.2	3.250	5.206	47.9
Cu70Zn30	80.3	5.151	3.402	4.726	81.7	19.7	3.249	5.206	47.9
Cu60Zn40	79.4	5.146	3.402	4.724	81.6	30.6	3.251	5.205	48.0
Cu87Zn10Co3	100	5.153	3.399	4.735	81.8	--			
Cu85Zn9Co6	100	5.150	3.425	4.737	81.8	--			
Zn100	--					100	3.249	5.205	47.9

**Table 3:** Average dimensions of particles and aggregates of the synthesized samples. Dim1 = dimension 1, Dim2 = dimension 2, Aggr. Range = aggregate size.

Sample	Dim 1	Dim2	Aggr. Range	Shape
Cu100	20.3 ± 5.2 nm	--		Nanoparticles
Zn100	50.2 ± 5.1 nm	250 ± 15 nm		Filament
Co100	20.1 ± 5.3 nm	--		Nanoparticles
Cu90Zn10	20.3 ± 9.9 nm			Nanoparticles
Cu80Zn20	27.0 ± 5.6 nm		205–250 nm	Hydrangea-like
Cu70Zn30	29.9 ± 6.0 nm		140–295 nm	Hydrangea-like
Cu60Zn40	32.6 ± 6.2 nm		140–230 nm	Hydrangea-like
Cu87Zn10Co3	23.4 ± 5.8 nm		110–280 nm	Hydrangea-like
Cu85Zn9Co6	28.3 ± 5.4 nm		140–280 nm	Hydrangea-like

**Table 4:** Atomic percentages of the synthesized oxides, obtained by EDX measurements. The standard deviation is 0.1% for all elements.

Sample	Cu at%	Zn at%	Co at%	O at%
Cu100	49.9	0.0	0.0	49.9
Zn100	0.0	49.7	0.0	50.1
Co100	0.0	0.0	42.7	57.1
Cu90Zn10	44.9	5.2	0.0	49.8
Cu80Zn20	39.7	9.8	0.0	50.2
Cu70Zn30	34.8	14.6	0.0	50.2
Cu60Zn40	29.9	19.8	0.0	50.0
Cu87Zn10Co3	43.5	5.0	1.6	49.8
Cu85Zn9Co6	42.3	4.3	3.1	50.1

**Table 5:** Literature data on cell viabilities (IC_50_) for CuO, ZnO, Co, CoO, Co_3_O_4_ and Cu_0.88_Zn_0.12_O NPs compared with the samples Cu100, Cu90Zn10 and Cu87Zn10Co3, taking into account NP dimensions, shape, cell lines, exposure dose and incubation time.

NPs	Dimension (nm)	Shape	Cell Line	Conc. Range (μg/mL)	Incubation Time	IC_50_ (μg/mL)	Ref.
CuO *	~55	Spherical	HEK 293	0–1000	24 h	410	[98]
CuO *	~40	Spherical	A 549	0–80	18 h	<40	[108]
	~3000	Spherical	A 549	0–80	18 h	>80	[109]
CuO	~55	Spherical	HEK 293	0–300	48 h	65.5	[96]
CuO	<50	Spherical	HEK 293	0–25	24 h	2.5	[97]
	<50	Spherical	MRC 5	0–25	24 h	~1.25	[97]
	<50	Spherical	HeLa	0–25	24 h	~1.25	[97]
	<50	Spherical	HCT 116	0–25	24 h	5	[97]
CuO	~12	Spherical	A 549	0–100	24 h	~19	[93]
	~12	Spherical	Caco2	0–100	24 h	~13	[93]
	~12	Spherical	Balb/c 3T3	0–100	24 h	~18	[93]
CuO	50–70	Rods	HepG2	0–100	24 h	85	[110]
	50–70	Rods	SK-Hep 1	0–100	24 h	25	[110]
ZnO	~90	Spherical	HEK 293	0–100	24 h	~25	[99]
	~90	Spherical	HEK 293	0–100	48 h	>50	[99]
ZnO	~110	Rods–spherical	HK 2	0–100	24 h	~20	[111]
ZnO *	20–1000	Irregular spherical	HEK 293	0–100	24 h	>100	[100]
	20–1000	Irregular spherical	MFC 7	0–100	24 h	~60	[100]
ZnO	>100	Spherical	HEK 293	0–50	24 h	~21	[101]
	>100	Spherical	HEK 293	0–50	48 h	~20	[101]
	>100	Spherical	HEK 293	0–50	72 h	~17	[101]
ZnO	~20	Rods–spherical	HEK 293	0–1000	24 h	~53	[102]
	~20	Rods–spherical	T98G	0–1000	24 h	~17	[102]
	~20	Rods–spherical	KB	0–1000	24 h	~38	[102]
ZnO	25–40	Spherical	HEK 293	0–100	3 h	>100	[103]
	25–40	Spherical	HEK 293	0–100	24 h	>75	[103]
	25–40	Spherical	HEK 293	0–100	48 h	>50	[103]
ZnO	~20	Spherical	A 549	0–100	24 h	~25	[93]
	~20	Spherical	Caco2	0–100	24 h	~30.	[93]
	~20	Spherical	Balb/c 3T3	0–100	24 h	~12	[93]
Co	~28	n.d.	HDMEC	0–50	24 h	>50	[112]
CoO	~62	Spherical	lymphocyte	0–50	24 h	~25	[113]
Co	~25	Spherical–polygonal	A 459	0–100	24 h	~93	[94]
CoO	~43	Spherical	A 459	0–100	24 h	inactive	[94]
Co_3_O_4_	~51	Clusters	A 459	0–100	24 h	inactive	[94]
Co_3_O_4_	~22	Hexagonal	A 549	0–40	24 h	inactive	[104]
	~22	Hexagonal	BEAS-2B	0–40	24 h	>40	[104]
Co_3_O_4_	~47	Polygonal	HepG2	0–100	24 h	inactive	[114]
	~47	Polygonal	Caco 2	0–100	24 h	inactive	[113]
	~47	Polygonal	HepG2	0–100	24 h	inactive	[113]
	~47	Polygonal	A 549	0–1000	24 h	~409	[113]
Co_3_O_4_	~9	Polygonal	A 549	0–100	24 h	~131	[93]
	~9	Polygonal	Caco2	0–100	24 h	~123	[93]
	~9	Polygonal	Balb/c 3T3	0–100	24 h	~135	[93]
Cu_0.88_Zn_0.12_O	2–10	Aggregate	HepG2	0–50	48 h	~10	[105]
	2–10	Aggregate	Bel7402	0–60	48 h	~35.6	[105]
	2–10	Aggregate	A 549			~24.4	[105]
	2–10	Aggregate	Panc28	0–10	48 h	~9.2	[105]
	2–10	Aggregate	HT1080	0–20	48 h	~25.8	[105]
	2–10	Aggregate	HELA	0–20	48 h	~6.5	[105]
	2–10	Aggregate	HUVEC	0–200	48 h	~148.6	[105]
	2–10	Aggregate	LO2	0–250	48 h	~294.94	[105]
Cu100	~20	Spherical	HEK 293	0–400	24 h	~87 **	This work
Cu90Zn10	~20	Spherical	HEK 293	0–400	24 h	~76 **	This work
Cu87Zn10Co3	~50	Hyrdangea	HEK 293	0–400	24 h	~68 **	This work

* biosynthesis, ** estimated using AAT Bioquest IC50 Calculator [94].

## Data Availability

Data are available upon request.
